# Fate determination in mesenchymal stem cells: a perspective from histone-modifying enzymes

**DOI:** 10.1186/s13287-015-0018-0

**Published:** 2015-03-19

**Authors:** Biao Huang, Gang Li, Xiao Hua Jiang

**Affiliations:** Key Laboratory for Regenerative Medicine, Ministry of Education, Epithelial Cell Biology Research Centre, School of Biomedical Sciences, Lo Kwee-Seong Integrated Biomedical Sciences Building, Shatin, New Territories, Hong Kong, PR China; Department of Orthopaedics & Traumatology, Li Ka Shing Institute of Health Science, Prince of Wales Hospital, 30-32 Ngan Shing Street, Shatin, New Territories, Hong Kong, PR China; Lui Che Woo Institute of Innovative Medicine, Faculty of Medicine, The Chinese University of Hong Kong, Hong Kong, SAR China; School of Biomedical Sciences Core Laboratory, The Chinese University of Hong Kong Shenzhen Research Institute, Shenzhen, 518057 China

## Abstract

Mesenchymal stem cells (MSCs) hold great promise for therapeutic use in regenerative medicine and tissue engineering. A detailed understanding of the molecular processes governing MSC fate determination will be instrumental in the application of MSCs. Much progress has been made in recent years in defining the epigenetic events that control the differentiation of MSCs into different lineages. A complex network of transcription factors and histone modifiers, in concert with specific transcriptional co-activators and co-repressors, activates or represses MSC differentiation. In this review, we summarize recent progress in determining the effects of histone-modifying enzymes on the multilineage differentiation of MSCs. In addition, we propose that the manipulation of histone signatures associated with lineage-specific differentiation by small molecules has immense potential for the advancement of MSC-based regenerative medicine.

## Introduction

Mesenchymal stem cells (MSCs) have been emerging as an extremely promising therapeutic agent for tissue regeneration and diseases largely because of their multi-potent properties and capacity for self-renewal. Stem cell self-renewal and differentiation require selective activation or silencing of specific transcription programs in response to environmental cues. This is achieved by intensive crosstalk between transcription factors and epigenetic modulators regulating the chromatin conformation that affects access of the transcriptional machinery to specific gene promoters. In contrast to growing information concerning transcriptional regulation, the epigenetic mechanisms governing MSC identity and fate determination are not well understood and remain an active area of investigation.

Within the context of chromatin, numerous histone-modifying enzymes reciprocally collaborate to establish and maintain a heritable epigenetic code by addition or removal of an array of covalent modifications in the core histones and other chromatin proteins. These modifications regulate gene expression as well as other genomic functions, and have been implicated in the defining of cell identity and fate. In this review, we summarize our current understanding of how histone-modifying enzymes modulate multi-lineage differentiation of MSCs. In addition, we discuss how an advanced understanding of epigenetic regulatory mechanisms will provide novel avenues for MSC-based therapy.

## Histone modification and histone-modifying enzymes

Epigenetic mechanisms play a pivotal role in the promotion of divergent transcriptional pathways during both embryonic development and adult tissue maintenance. Regulation of gene expression at the epigenetic level occurs via modifications of chromatin architecture by facilitating the opening of DNA (euchromatin) to permit transcription, or the condensing of DNA (heterochromatin) to repress transcription [1]. Therefore, the architecture of chromatin is essential for the regulation of various chromatin-based cellular processes, and is dynamically modulated through the orchestration of multiple mechanisms, including histone modification, DNA methylation, chromatin remodeling, and non-coding RNA. Among these mechanisms, post-translational histone modifications, such as methylation, acetylation, phosphorylation, ADP-ribosylation and ubiquitination, play a central role and have been extensively studied over the past two decades. These modifications are brought about by a series of ‘writing’ and ’erasing’ events executed by histone-modifying enzymes [2]. Histone-modifying enzymes collaborate to catalyze the addition or removal of an array of covalent histone modifications, which subsequently function as a ‘histone code’ that would be recognized by chromatin effector molecules (‘reader’), causing the recruitment of other molecules to alter the chromatin and/or transcription states [2,3]. Various groups of histone-modifying enzymes supplement (writer) or eliminate (eraser) covalent modifications to histone proteins. For instance, histone methyltransferases (HMTs) and histone acetyltransferases (HATs) supplement methyl and acetyl groups, respectively, whereas histone demethylases (HDMs) and histone deacetylases (HDACs) remove methyl and acetyl groups, respectively. The discovery of these enzymes represents a milestone in understanding the biological functions underlying histone modifications because they provide direct evidence linking histone conformation states and transcriptional regulation. The categories, specificity and mechanisms of various histone-modifying enzymes have been extensively reviewed elsewhere [4-6]. In this review, we mainly focus on the role of histone-modifying enzymes in the regulation of MSC multi-lineage differentiation, with emphasis on histone acetylation, histone methylation and their corresponding histone-modifying enzymes. A list of histone writers, erasers and readers and their corresponding catalytic sites is provided in Table 1.

**Table 1:** **Epigenetic readers, writers and erasers**

**Family**	**Activity**	**Major catalytic site**	**Major classes**	**Representative member**	**Classic inhibitors**
**Writers**					
Histone acetyltransferases	Catalyze histone acetylation	H3K9/K14/K56, H4K5/K8/K16, H2AK5	(1) Gcn5/PCAF	P300/CBP, Tip60, Gcn5	Acetyl-CoA derivatives, anacardic acid, curcumin
			(2) MYST		
			(3) p300/CBP		
			(4) Rtt109		
Histone methyltransferases	Catalyze histone methylation	H3K4/K9/K27/K36/K79, H4K20, H3R8	(1) SUV39	DOT1L, EZH2, SUV39H1	EPZ00477, GSK343, UNC1999
			(2) SET1		
			(3) SET2		
			(4) RIZ		
			(5) PRMTs		
**Erasers**					
Histone deacetylases	Catalyze histone deacetylation	H3K9/K14, H4K5/K12/K8	(1) HDAC I	HDAC1, HDAC3, HDAC6	TCA, vorinostat, romidepsin
			(2) HDAC II		
			(3) HDAC III		
			(4) HDAC IV		
Histone demethylases	Catalyze histone demethylation	H3K4/K9/K27/K36/K79, H4K20	(1) Lysine-specific demethylases	JMJD2A, KDM5B, KDM2A	Tranylcypromine, GSK-J1, 8-hydroxyquinolines
			(2) Jumonji domain-containing demethylases		
**Readers**					
Bromodomain-containing proteins	Binding the acetylated lysine residue	H3K14, H4K5/K8/K16	Bromodomains	GCN5, Brdt, Rsc4	JQ1, GSK2801
PHD-containing proteins	Binding the methylated lysine residue, or the acetylated lysine residue	H3K4/K9/K14	PHD domains	RAG2, BHC80, TAF3	-
Methyl-lysine- and/or methyl-arginine-binding domain-containing proteins	Binding the methylated lysine residue, or the methylated arginine residue	H3K4/K9/K23/K27/K36/K79, H4K20, H1K26, H3R17, H4R3	(1) Tudor domains	53BP1/Crb2, HP1, PHF20L1	UNC669, UNC1215
			(2) MBT domains		
			(3) Chromodomains		
			(4) PWWP domains		

HDAC, histone deacetylase; PRMT, protein arginine methyltransferases; PWWP domain, Pro-Trp-Trp-Pro motif; MBT domain, malignant brain tumor domain; PHD domain, Cys_4_-His-Cys_3_ motif.

## Histone-modifying enzymes regulate mesenchymal stem cell multi-lineage differentiation

MSCs, also referred to as multipotent stromal cells or mesenchymal stromal cells, are one of the most intensively studied adult stem cell types, holding significant promise for regenerative therapies. At present, a large number of studies are focusing on identifying extrinsic regulators and their intrinsic target transcription factors that regulate MSC properties and functions, whereas very little is known regarding the epigenetic events that control MSC identity and/or functions. Indeed, accumulating evidence indicates that cell fate and function are determined by DNA-binding transcription factors that are regulated more specifically at the epigenetic level, as we learned from pluripotent stem cells such as embryonic stem (ES) cells and induced-pluripotent stem cells [7,8]. Furthermore, ES cells have been widely used as a model to decipher the epigenetic mechanisms of cell fate determination and cell homeostasis. The pluripotent capacity of ES cells correlates with the requirements of dynamic genomic organization to support their functional plasticity. Genes that are involved in maintaining both repressive (H3K27me3) and activating (H3K4me3) histone modifications are important in ES cells for early lineage commitment. These bivalent genes, which encode mainly transcriptional factors involved in lineage specification, such as Sox, Fox, Pax, Irx, and Pou gene family members, are considered to be poised for rapid activation in response to appropriate differentiation signals [9,10].

Like ES cells, several adult tissues, including sperm, testis, cerebellum, and the hematopoietic compartment, have been reported to contain bivalent chromatin domains as well [11-13]. For instance, during hematopoietic stem cell differentiation, most genes associated with bivalent chromatin states remain silent and lose the H3K4me3 mark after differentiation. In contrast, genes without the H3K27me3 mark are associated with increased levels of H2A.Z, H3K4me1, H3K9me1, H4K20me1 and RNA polymerase II, and become activated after differentiation [11]. Thus, it is plausible that many chromatin regulatory factors that have been identified to control ES cell fates play similar roles in adult stem cells. For MSCs, while it is still unclear whether chromatin structure and histone-modifying enzymes utilize similar mechanisms to modulate gene expression, emerging evidence indicates that histone modifications play an important role in the control of MSC differentiation. Distinct histone-modifying enzymes associated with MSC lineage conversion are listed in Table 2.

**Table 2:** **Distinct histone-modifying enzymes associated with multi-lineage differentiation of mesenchymal stem cells**

**Histone-modifying enzymes**	**Abbreviation**	**Catalytic activity**	**Catalytic specificity**	**Proposed functions**	**References**
**Histone methylation**					
Enhancer of Zeste homology 2	EZH2	HMT	H3K27me3	Promoting adipogenesis	[17,23,41]
				Inhibiting neurogenesis	
				Inhibiting osteogenesis	
Lysine demethylase 6A	KDM6A	HDM	H3K27me3	Promoting osteogenesis	[23]
ERG-associated protein with a SET domain	ESET (SETDB1)	HMT	H3K9me2/3	Promoting adipogenesis	[15,18]
				Inhibiting osteogenesis	
Lysine demethylase 4B	KDM4B	HDM	H3K9me3	Promoting osteogenesis	[22]
Lysine demethylase 6B	KDM6B	HDM	H3K27me3	Promoting osteogenesis	[22]
Plant homeodomain finger 2	Phf2	HDM	H3K9me2	Promoting adipogenesis	[21,24,29]
				Promoting chondrogenesis	
Lysine demethylase 2A	KDM2A (FBXL11)	HDM	H3K36me2	Inhibiting osteo/dentinogenesis	[27,28]
			H3K4me3		
PR-Set7 (SETD8, SET8, KMT5A)	PR-Set7/Setd8 (SET8)	HMT	H4K20me1	Promoting adipogenesis	[20]
Mixed-lineage leukemia H3K4 methyltransferase protein	MLL3/MLL4	HMT	H3K4me3	Promoting adipogenesis	[19]
Lysine-specific demethylase 1	LSD1	HDM	H3K4/K9	Promoting adipogenesis	[18]
**Histone acetylation**					
Histone deacetylases 2	HDAC2	HDAC	H3K9/K14ac	Modulating osteogenesis	[37,46]
				Inhibiting cardiomyogenesis	
Histone deacetylases 1	HDAC1	HDAC	H3K9/K14ac	Inhibiting adipogenesis	[37,38,46]
				Inhibiting cardiomyogenesis	
Histone deacetylases 6	HDAC6	HDAC	H3K9/K14ac/H4K8ac	Promoting adipogenesis	[39]
CREB binding protein and p300	CBP/p300	HAT	H3K9/K14/K56ac	Promoting chondrogenesis	[32,33,40]
			H4K5/K8ac	Promoting adipogenesis	
Tat-interactive protein 60 kDa	Tip60	HAT	H2AK5ac	Promoting adipogenesis	[34,35]
General control nonrepressed protein 5	Gcn5	HAT	H3K9/K14ac	Accelerating cardiomyocyte differentiation	[45]
			H4K8/K16ac		

HAT, histone acetyltransferase; HDAC, histone deacetylase; HDM, histone demethylase; HMT, histone methyltransferase.

### Tri-lineage differentiation of mesenchymal stem cells

#### Histone methyltransferases and histone demethylases

**Figure 1:**
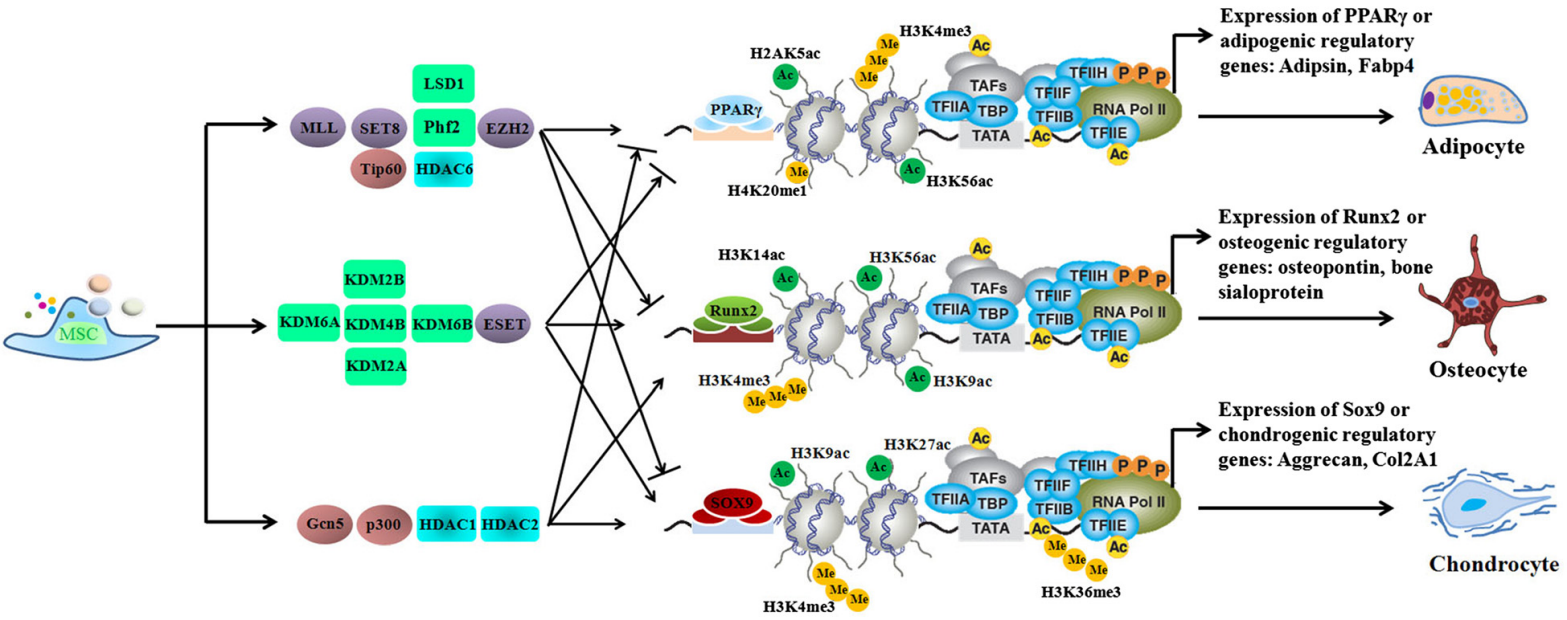
**A variety of histone-modifying enzymes are involved in the dynamic regulation of mesenchymal stem cell (MSC) differentiation into adipocytes, osteocytes or chondrocytes.**

MSCs can be readily differentiated into osteoblasts, adipocytes or chondrocytes, while directed differentiation strictly relies on an orchestrated balance among these three lineages. Growing evidence suggests that, upon differentiation of MSCs, various histone modifications change to facilitate the activation or repression of key transcription factors, guiding development towards specified cell lineages (Figure 1). Among these modifications, histone methylation is crucial for chromatin reorganization and regulation of gene transcription. For example, lysine (K) methylation at H3K9, H3K27, and H4K20 is associated with transcriptionally silenced chromatin, while methylation at H3K4, H3K36, and H3K79 is correlated with transcriptionally active regions. The dynamic alteration of histone methylation during lineage commitment is achieved by the reciprocal action of HMTs and HDMs.

Furthermore, histone methylation has a role in osteogenic differentiation from MSCs. It has been shown that *HoxA10*, a gene necessary for the embryonic patterning of skeletal elements, contributed to osteogenic lineage determination through enrichment of H3K4me3 at the promoter region [14]. Increased HoxA10 activated Runx2, alkaline phosphatase and osteocalsin (OC) during osteogenic differentiation [14]. While this study did not identify the specific HMTs responsible for the enrichment of H3K4Me3, our work provided a direct link between histone methylation and transcriptional activation of master osteogenic transcription factors. Direct evidence showing the role of HMT in osteogenesis is provided by another study demonstrating that ESET (also known as SETDB1, a HMT methylating histone H3 at lysine 9) regulates the osteogenic differentiation of MSCs during postnatal bone development [15]. Overexpression of ESET deregulates Runx2 and Indian hedgehog (Ihh), both well known for their critical roles in the osteogenic and chondrogenic differentiation of MSCs. The function of PcG (polycomb group) proteins in osteogenic differentiation of MSC has also been investigated. Additionally, PcG proteins form multi-protein complexes (termed polycomb-repressive complexes, PRCs) to regulate chromatin structure and dynamically change gene expression during differentiation [16]. Genome-wide investigation of EZH2 (one of the subunits of PRC2, which specifically trimethylates H3K27me3) target genes showed that over 4,000 genes bound EZH2 before differentiation, whereas less than 30 genes were bound after osteogenic differentiation with a concomitant decrease in the H3K27me3 mark [17]. In addition, the study reported that activation of cyclin-dependent kinase 1 (CDK1) promoted osteogenic differentiation through disruption of the PRC2, which subsequently led to the activation of Runx2 expression during osteogenesis. Altogether, this study suggests that the EZH2-mediated decrease in H3K27me3 binding during osteogenic differentiation might favor the activation of lineage-specific genes. It is to be noted that several HMTs have been reported to regulate adipogenesis. For instance, knockdown of SETDB1, a H3K9 methyltransferase, promoted adipogenic differentiation by decreasing H3K9me2 and increasing H3K4me2 levels [18]. It was also reported that MLL3/MLL4 (methyltransferases of histone H3 Lys4) were involved in the establishment of an ASCOM complex (activating signal cointegrator-2 complex), which directly interacted with peroxisome proliferator-activated receptor (PPAR)-γ and promoted adipogenesis [19]. This idea was further supported by findings that mice expressing inactivated MLL3 exhibited decreased adipose tissue [19]. Apart from that, PR-Set7/Setd8, the histone H4 Lys20 (H4K20) monomethyltransferase, was shown to modulate adipogenesis through PPAR-γ and its partner protein, retinoid X receptor-α, via a positive feedback loop [20].

Recently, distinct HDMs have been identified as critical regulators of MSCs differentiation [18,21-24]. During adipocyte differentiation, expression of the H3K4/K9 demethylase LSD1 was upregulated and knockdown of LSD1 resulted in impaired adipocyte differentiation of 3 T3-L1 preadipocytes by decreasing H3K4me2 levels while increasing H3K9me2 levels at the promoter region of the CEBPα gene [18]. Another H3K9me2 demethylase, Phf2, was recently identified as a positive regulator in adipogenesis by coactivating both C/EBPα and C/EBPΔ [21,24]. The critical role of H3K9 demethylation has been further validated by a study showing mice carrying a disruption in the gene encoding Jhdm2a, another H3K9 demethylase, exhibit obesity and hyperlipidemia [25]. On the other hand, it was shown recently that the histone demethylases KDM4B and KDM6B play critical roles in osteogenic commitment of MSCs by removing H3K9me3 and H3K27me3. Depletion of KDM4B or KDM6B significantly reduced osteogenic differentiation and increased adipogenic differentiation. Mechanistically, while KDM6B was required for the expression of HOX genes by removing H3K27me3, KDM4B regulated the expression of DLX genes by removing H3K9me3 [22]. This study provides convincing evidence that HDMs play vital roles in governing lineage-specific decisions during MSC differentiation. Following this study, Hemming and colleagues [23] further revealed that the reciprocal action of EZH2 and KDM6A could coordinate to achieve an epigenetic switch in human MSC differentiation. EZH2 specifically trimethylated H3K27me3, whereas KDM6A removed the methyl group from H3K27me3. Consequently, while inhibition or knockdown of EZH2 resulted in decreased adipogenesis and increased osteogenesis, knockdown of KDM6A led to increased adipogenesis and decreased osteogenesis. Of great importance, EZH2 and KDM6A affected the same group of master regulatory genes involved in adipogenesis and osteogenesis, including those encoding PPARγ, CEBPα, Adipsin, Runx2, OC and osteopotin [23]. These findings demonstrate that epigenetic shifts centered on H3K27me3 determine MSC fate via coordinated modification by both HMTs and HDMs. The importance of HDMs in MSC osteogenic differentiation has been further demonstrated in another study investigating the molecular basis of a rare human genetic disease, oculo-facial-cardio-dental syndrome [26]. It was reported that BCL-6 co-repressor (BCOR) mutation increased histone H3K4 and H3K36 methylation via KDM2A in MSCs, thereby reactivating transcription of AP-2α, leading to increased osteo-dentinogenic potential of MSCs in oculo-facial-cardio-dental syndrome patients [27]. KDM2A is also involved in other lineage differentiation of MSCs. Depletion of KDM2A enhanced the adipogenic and chondrogenic differentiation potential by upregulating SOX2 and NANOG [28]. Compared with osteogenic and adipogenic differentiation, few published studies have reported on epigenetic regulation of chondrogenic differentiation from MSCs. A recent study using a mouse model revealed that AT-rich interactive domain 5b (Arid5b) functioned as a transcriptional co-regulator of Sox9 and recruited Phf2 to the promoter region of Col2a1 and aggrecan, promoting the demethylation of H3K9me2 specifically in these genes [29].

#### Histone acetyltransferases and histone deacetylases

Histone acetylation is one of the most abundant and dynamic histone modifications. The actual level of histone acetylation is dependent on highly orchestrated interplay between HATs and HDACs. Numerous studies have reported the involvement of histone acetylation in regulating lineage-specific gene expression in MSCs. Shen and colleagues [30] observed that both the promoter and coding regions of the OC gene contained low levels of acetylation of histone H3 and H4 during the proliferative period of osteogenic differentiation when this gene was inactive. However, OC became active in the mature osteoblasts, which correlated with enriched H4 acetylation, providing the link between osteogenic differentiation and histone acetylation. Subsequently, ChIP-on-chip analysis revealed that H3K9Ac correlated with activating genes and H3K9me2 was associated with silencing genes in osteogenic differentiation from MSCs. This study also found that many vitamin D receptor elements were at the gene promoters in both H3K9Ac-decreased and H3K9me2-increased groups, suggesting that the vitamin D receptor might be a potential regulator mediating deacetylation and dimethylation of H3K9 during osteogenic differentiation [31]. Histone acetylation is also associated with chondrocyte differentiation. It has been reported that several transcription factors and co-activators, such as Scleraxis/E47 and p300, cooperatively modulate Sox9-dependent transcription by interacting with Sox9. The Sox9- related transcriptional apparatus activates its target gene expression through p300- mediated histone acetylation on chromatin during chondrogenesis [32,33]. In addition, HATs p300/CBP and Tip60 have been shown to promote the expression of adipogenic genes through direct interaction with PPAR-γ [34,35]. Specifically, the two homologous cofactors p300 and CBP bind to the amino terminus of PPARγ2 in a ligand-independent manner, leading to further recruitment of HATs that appropriately modify the surrounding chromatin, allowing the transcriptional machinery access to the gene promoter [36]. Thus, these studies in cultured cells have shown that lineage-associated core transcription factors interact with HATs, which stimulate transcription by acetylating nucleosomal histones, thereby relaxing the chromatin structure and facilitating transcription.

On the other hand, HDACs, a conserved family of chromatin-modifying enzymes that repress transcription by deacetylating nucleosomal histones, are also associated with key transcription factors, counteracting the functions of HATs. For instance, through the systematic genetic deletion of HDAC genes in cultured mesenchymal precursor cells, Haberland and colleagues showed that deletion of HDAC1 and HDAC2 led to reduced lipid accumulation, validating the redundant and requisite roles of class I HDACs in adipogenesis [37]. The regulatory role of class I HDAC in lineage commitment was further strengthened by the recent finding that HDAC1 specifically occupied the −1,286 to −1,065 bp promoter region of the C/EBPα gene during adipogenesis [38]. In contrast, HDAC6, a representative of class II HDACs, has been shown to promote adipogenesis, yet inhibits osteogenesis in human adipose tissue-derived MSCs. Overexpression of miR-22, which directly targets HDAC6, repressed the expression of adipogenic transcription factors whereas it upregulated the expression of osteogenic genes, indicating a positive role of HDAC6 in adipogenesis [39]. Of particular interest, it has been observed recently that HATs and HDACs are involved in the regulation of the same transcription factor through the modification of histones, and also the regulation of each other during lineage commitment. During chondrogenesis, YY1 and p300 competitively bind to the core promoter region of ChM-I (the chondromodulin-I gene). In particular, YY1 (a transcriptional repressor) establishes and maintains transcriptional silencing by recruiting HDACs while p300 promotes the expression of ChM-I through cooperation with Sp3 [40], indicating HAT and HDAC can compete for the same promoter region of lineage-associated genes.

### Trans-lineage differentiation of mesenchymal stem cells

**Figure 2:**
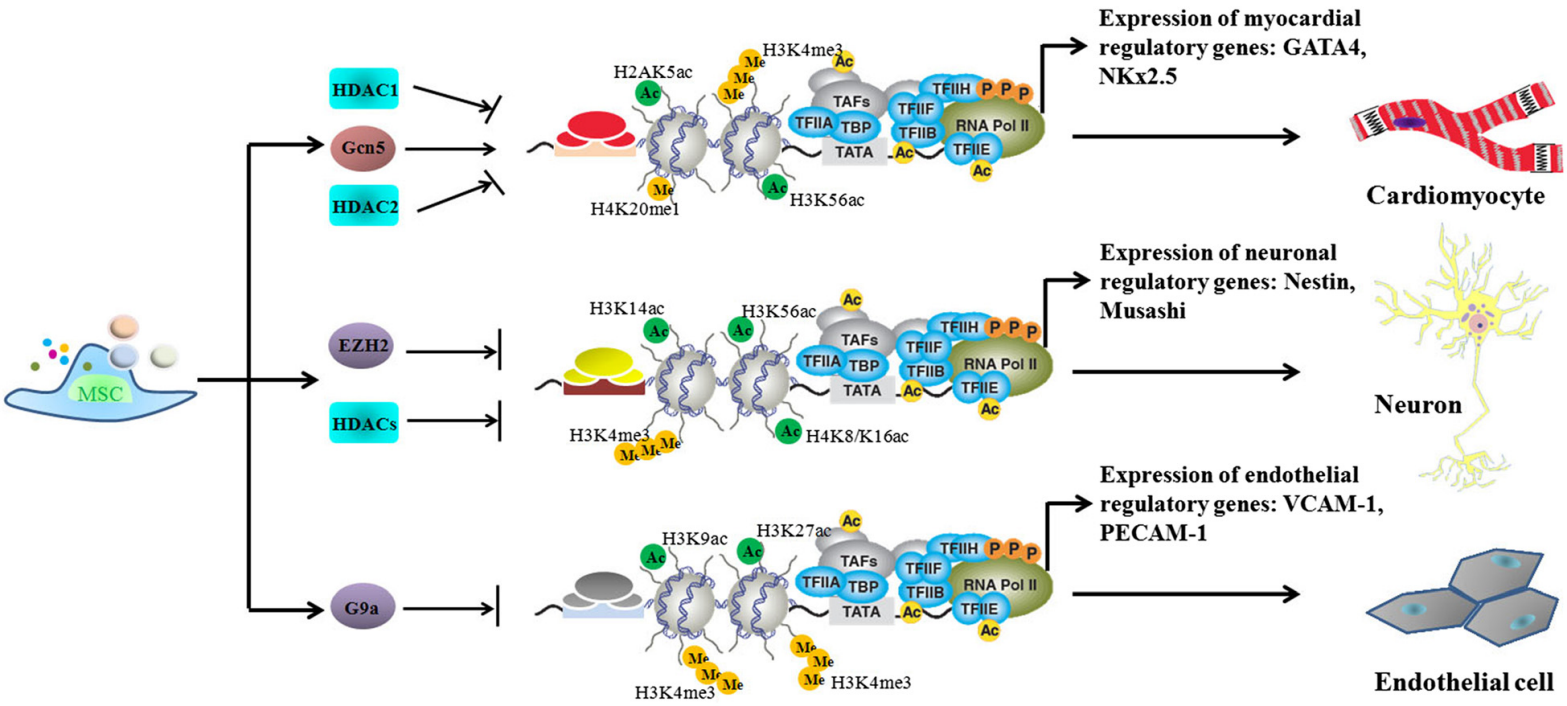
**Several histone-modifying enzymes participate in the regulation of trans-lineage differentiation of mesenchymal stem cells (MSCs).**

MSCs have the ability to cross oligolineage boundaries and differentiate into different kinds of cell types, such as neurons, cardiomyocytes, hepatocytes and endothelial cells, under appropriate culture conditions, indicating that they are more plastic than we previously thought. While the molecular mechanisms underlying MSC transdifferentiation are largely unknown, it is plausible that epigenetic plasticity is one of the major factors contributing to this unique feature of MSCs. Indeed, increasing evidence has shown that both histone methylation and acetylation are involved in the regulation of MSC transdifferentiation (Figure 2). For example, during the neuronal induction of MSCs, EZH2 negatively regulates neuronal differentiation by binding to the promoter region of PIP5K1C to suppress its transcription. Knockdown of EZH2 activates PIP5K1C and increases the intracellular Ca^2+^ level via the PI3-kinase/Akt signaling pathway, which ultimately leads to the neuronal differentiation of MSCs both *in vitro* and *in vivo* [41]. Interestingly, HDACs are also involved in the neurogenesis of MSCs, with a HDAC inhibitor such as valproic acid significantly stimulating the expression of the neural progenitor markers Nestin, Musashi, CD133, and GFAP in bone marrow-derived MSCs, promoting neuronal differentiation [42]. Valproic acid can also considerably improve the hepatic differentiation of bone marrow-derived MSCs [43]. Histone modifiers also control the differentiation of MSCs into mesoderm cell lineages. During 5-azacytidine-induced cardiomyocyte differentiation, Gcn5 (a HAT) increases the level of histone acetylation on the promoter regions of the early cardiomyocyte-specific genes GATA4 and NKx2.5, and accelerates differentiation [44]. In contrast, suppression of HDAC1 or HDAC2 by small interfering RNAs enhances acH3 and acH4 levels and upregulates cardiac-specific gene expression in MSCs [45]. Moreover, a recent report showed that the epigenetic modifying drug BIX-01294 (a histone G9a methyltransferase inhibitor) improved endothelial differentiation of adipose tissue-derived MSCs through upregulation of several endothelial markers and factors associated with blood vessel formation, such as VCAM-1, PECAM-1, von Willebrand factor, and VEGFR-2 [46]. Taken together, these studies provide a deeper insight into the epigenetic mechanisms controlling MSC fate determination, and suggest molecular models of how key lineage-associated transcriptional factors are linked to histone modifications in MSCs.

## Development of small molecules targeting histone-modifying enzymes

The knowledge obtained from epigenetic studies on MSC differentiation could be applied to regenerative medicine. Indeed, it is conceivable that manipulation of the epigenetic signatures associated with lineage-specific differentiation can direct patient-specific therapy. For instance, as chemically modifiable enzymes, KDM4B and KDM6B could be activated or deactivated to regulate specific lineage decisions of MSCs, thereby holding promising potential as therapeutic targets for stem cell-mediated regenerative medicine as well as the treatment of human metabolic diseases such as osteoporosis and obesity [22]. In this regard, screening and development of small molecules specifically targeting histone-modifying enzymes could be a feasible strategy. Indeed, many chemicals targeting HATs or HDACs have been developed and have undergone clinical trials [47]. In particular, various HDAC inhibitors have been used in various clinical trials to treat different cancers and have shown promising results alone or in combination with conventional oncological modalities [48]. Furthermore, drugs targeting HMTs and HDMs are being developed. For example, a specific inhibitor of Suv39 HMT was identified [49] and, more recently, GSK 126, which inhibits EZH2, was developed to suppress the growth of lymphoma [50]. In general, a more defined understanding of the epigenetic mechanisms underlying MSC fate determination would be essential for the development of small molecules and their ultimate application in regenerative medicine. On the other hand, it would be essential to evaluate the adverse effects of such molecules because almost all histone-modifying enzymes are ubiquitously expressed regardless of tissue or cell type.

## Conclusion

It is quite apparent that significant progress has been made during the past few years in identifying the histone-modifying enzymes involved in the fate determination processes of MSCs. These functional enzymes fine-tune epigenetic environments at regulatory regions of multiple transcriptional factors and are closely related to gene activation/repression. The challenge for the future is to gain more insights into the global epigenetic regulatory mechanisms governing the commitment of MSCs to specified lineages. In this regard, research into MSCs lags far behind that of pluripotent stem cells since a very limited number of studies have been designed to determine epigenetic signatures during MSC lineage commitment. While one analysis of genes regulating adipogenic differentiation of human MSCs has revealed dynamic changes in histone marks reminiscent of those seen in ES cells [51], other studies have found that histone modifications were globally stable throughout differentiation but showed distinct and highly dynamic distribution patterns at specific genes, indicating that, unlike pluripotent stem cells, histone modifications in MSCs appear to be gene-specific [52,53]. Ideally, by using whole-genome chromatin immunoprecipitation and deep sequencing, defined studies will open up a new epigenetic landscape in MSCs. In addition, more comprehensive studies aiming to understand how various epigenetic elements compile and coordinate to achieve lineage commitment are necessary. Considering the fact that epigenetic research is paving the way for many new breakthroughs in the prevention, diagnosis and treatment of human diseases, focused efforts on the detailed mechanisms linking epigenetic regulatory mechanisms to adult stem cell function in physiological/pathological conditions have an immense potential for improving human health and welfare.

## Abbreviations

ES: Embryonic stem
HAT: Histone acetyltransferase
HDAC: Histone deacetylase
HDM: Histone demethylase
HMT: Histone methyltransferase
MSC: Mesenchymal stem cell
OC: Osteocalcin
PPAR: Peroxisome proliferator-activated receptor
PRC: Polycomb repressive complex
